# Combining pedigree and genomic information to improve prediction quality: an example in sorghum

**DOI:** 10.1007/s00122-019-03337-w

**Published:** 2019-04-09

**Authors:** Julio G. Velazco, Marcos Malosetti, Colleen H. Hunt, Emma S. Mace, David R. Jordan, Fred A. van Eeuwijk

**Affiliations:** 1 Department of Plant Breeding, National Institute of Agricultural Technology (INTA), EEA Pergamino, B2700WAA Pergamino, Argentina; 2 Biometris, Wageningen University and Research, 6700AA Wageningen, The Netherlands; 3 Queensland Alliance for Agriculture and Food Innovation, The University of Queensland, Hermitage Research Facility, Warwick, QLD 4370 Australia; 4 Department of Agriculture and Fisheries, Hermitage Research Facility, Warwick, QLD 4370 Australia

## Abstract

**Key message:**

The use of a kinship matrix integrating pedigree- and marker-based relationships optimized the performance of genomic prediction in sorghum, especially for traits of lower heritability.

**Abstract:**

Selection based on genome-wide markers has become an active breeding strategy in crops. Genomic prediction models can make use of pedigree information to account for the residual polygenic effects not captured by markers. Our aim was to evaluate the impact of using pedigree and genomic information on prediction quality of breeding values for different traits in sorghum. We explored BLUP models that use weighted combinations of pedigree and genomic relationship matrices. The optimal weighting factor was empirically determined in order to maximize predictive ability after evaluating a range of candidate weights. The phenotypic data consisted of testcross evaluations of sorghum parental lines across multiple environments. All lines were genotyped, and full pedigree information was available. The performance of the best predictive combined matrix was compared to that of models fitting the component matrices independently. Model performance was assessed using cross-validation technique. Fitting a combined pedigree–genomic matrix with the optimal weight always yielded the largest increases in predictive ability and the largest reductions in prediction bias relative to the simple G-BLUP. However, the weight that optimized prediction varied across traits. The benefits of including pedigree information in the genomic model were more relevant for traits with lower heritability, such as grain yield and stay-green. Our results suggest that the combination of pedigree and genomic relatedness can be used to optimize predictions of complex traits in crops when the additive variation is not fully explained by markers.

## Introduction

Selection based on dense genome-wide markers has become a revolutionary alternative to traditional genetic evaluations for improving quantitative traits in crops (Jannink et al. 2010; Crossa et al. 2017). This selection technique exploits the association between high-density markers and unknown causative genes to predict genetic merit or breeding value (BV). Genomic prediction (GP) is expected to increase accuracy of evaluations by capturing large and small allelic effects across the genome simultaneously (Meuwissen et al. 2001). These effects are estimated using phenotypic and genotypic data from a reference breeding population and then integrated to predict genome-assisted BVs of untested selection candidates that have been only genotyped. The implementation of this prediction method can potentially lead to higher rates of genetic gain and lower phenotyping costs compared to classical phenotypic or pedigree-based selection.

The underlying requirement for obtaining optimal genomic predictions is that available markers or haplotypes of markers are in complete linkage disequilibrium (LD) with quantitative trait loci (QTLs) of interest. Consequently, a major assumption is that the full additive genetic variance can be accurately explained by markers (Goddard 2009). When this condition is not met due to incomplete LD with causative genes, accuracy of prediction is expected to decline (Habier et al. 2007; Goddard et al. 2011). In such cases, pedigree information may be incorporated into GP models to account for the residual polygenic variance not captured by markers and to reduce empirical bias of predictions. Pedigree and genome-wide markers can offer different, yet complementary, information on genetic relatedness among individuals. While pedigrees represent expected average relationships describing potential transmission of genes, genomic data provide observed realized relationships. The latter are expected to be more accurate because markers can trace alleles, capturing random Mendelian sampling and unknown ancestral relationships not considered in the pedigree. Nevertheless, the inclusion of a residual genealogical effect in prediction models might account for potential LD patterns not explained by markers at population and family levels. Consequently, the joint use of pedigree and genomic information may provide better estimates of genetic similarities between genotypes, affecting predictive performance.

The benefits of exploiting pedigree and marker data in plants have been previously reported in the context of QTL and association mapping (Bink et al. 2002; Parisseaux and Bernardo 2004; Malosetti et al. 2007). In sorghum, Jordan et al. (2004) demonstrated that combining pedigree information with mapped markers facilitated the identification of genetic regions under selection in a breeding population. Within the GP framework, several approaches to combine genealogy with genomic data have been reported. Bayesian regression and semi-parametric models were firstly implemented in crops (de los Campos et al. 2009; Crossa et al. 2010). Alternative models based on best linear unbiased prediction (BLUP) have been used in maize and wheat (Albrecht et al. 2011; Burgueño et al. 2012; Sukumaran et al. 2017). These BLUP models include two mutually independent genetic effects, one depending on a pedigree-based relationship matrix (**A**) and one depending on a genomic relationship matrix (**G**). A similar approach based on BLUP integrates pedigree and genomic relatedness into a single matrix. This strategy has been applied in the context of animal genetic evaluations (VanRaden 2008; Goddard et al. 2011; Gao et al. 2012), but it has not been implemented so far in plants. Different models combining pedigree and genome-wide markers have also been proposed for specific situations where not all individuals in the reference population are genotyped (Legarra et al. 2014; Liu et al. 2014; Fernando et al. 2016).

Compatibility between **A** and **G** may be an issue when jointly used in prediction models. It is important to consider that genetic relationships in both matrices are in different scales with reference to the base population (Legarra et al. 2014). Therefore, the use of a compatible scale for pedigree and genomic relationships should be considered when estimating and interpreting genetic variances and derived measures, such as heritability and expected response to selection. Different scaling methods have been implemented to achieve the same base population for **A** and **G** (Vitezica et al. 2011; Christensen 2012). These corrections are aimed at accounting for the non-randomness of genotyping due to the previous selection decisions.

Sorghum [*Sorghum bicolor* (L.) Moench] is the fifth most important cereal crop worldwide after wheat, maize, rice and barley. Its drought tolerance ability makes it a strategic crop for sustainable grain production in the perspective of climate change and increasing food demand. Early stage breeding of hybrid sorghum involves the development of elite inbred lines, which will be subsequently used as parents of commercial hybrids. The initial selection of superior parental lines is typically based on their additive genetic values estimated from testcross performance trials. Genomic selection could be beneficial for reducing time and field testing resources during the testcross evaluation step, increasing efficiency of hybrid development. Despite the new opportunities offered by GP methods to accelerate genetic progress in sorghum, empirical studies are still limited compared to other crops (Kulwal 2016). A first implementation of GP in sorghum was reported for biomass traits in a global germplasm collection (Yu et al. 2016). In a more recent study by Hunt et al. (2018), genomic models were applied for prediction of testcross yield performance in the context of individual trial analysis.

For the present research, we considered a multi-year and multi-location testcross evaluation of sorghum parental lines using several testers and including different production and adaptability traits. The dataset belongs to a public breeding program in Australia where identification of superior parental lines is typically based on extensive phenotyping of progeny performance. This interesting case study was used to explore the potential of combining pedigree, markers and phenotypic data for optimization of genomic prediction in sorghum.

The objectives of this article were to explore the impact of combining pedigree and genomic information on the quality of BV predictions, and to determine the combinations of information that optimize predictive performance for different traits. For these purposes, we applied BLUP models using a blended kinship matrix constructed as a weighted combination of matrices **A** and **G**, where different weights were tried in search of improved prediction quality.

## Materials and methods

### Phenotypic data

The dataset is part of the sorghum breeding program for female parental lines conducted by the University of Queensland and the Department of Agriculture and Fisheries in Queensland, Australia. Female lines are evaluated in hybrid combination with different male testers across multiple environments. Testcross performance is then used to estimate the general combining ability (GCA) or BV of parental lines.

The phenotypic records used in this study consisted of 26 testcross performance trials where a total of 646 female lines were tested across 12 locations over a period of 7 years between 2008 and 2014. This trial series comprises a representative sample from a target population of environments covering the main sorghum cropping region in Australia. Phenotypes of 2645 testcross hybrids were used to assess female lines in crosses with one to five different testers. These male parents were chosen to express contrasting levels of yield potential and stay-green capacity (Jordan et al. 2012). In each trial, between 110 and 315 lines were evaluated and 3–5 testers were used. Across the dataset, more than 50% of the lines were crossed with at least three different testers and grown in at least nine environments. The sets of testcrosses entering evaluation were designed to provide a degree of connectivity between lines and testers across trials. Each experiment was laid out as a resolvable partially replicated design (Cullis et al. 2006), where 30% of the testcross hybrids had two replicates and commercial varieties were included with additional replication. The number of testcrosses in each trial varied between 247 and 858. We considered four productivity and adaptability traits routinely measured by the program: grain yield (GY), stay-green (SG), plant height (PH) and flowering time (FT). Stay-green is a drought resistance trait that expresses as delayed leaf senescence in environments where water-stress conditions occur (Borrell et al. 2014). In this dataset, the stay-green trait was expressed in nine trials and observations were available for 603 lines.

### Pedigree and genotypic data

Inbred parent lines were derived from pedigree breeding methods resulting in a highly structured breeding population. The 646 female lines are basically grouped into 74 full-sib families including different numbers of siblings. Genealogical information on the tested lines and 499 ancestors tracing back 28 generations was available to compute the pedigree-based relationship matrix **A**.

All the female lines were genotyped using an integrated DArT and genotyping-by-sequencing (GBS) methodology involving complexity reduction in the genomic DNA to remove repetitive sequences using methylation sensitive restriction enzymes prior to sequencing on next generation sequencing platforms (DArT, www.diversityarrays.com). The sequence data generated were then aligned to the most recent version (v3.1.1) of the sorghum reference genome sequence (Paterson et al. 2009) to identify single-nucleotide polymorphism (SNP) markers. SNPs with minor allele frequency lower than 2.5% or more than 20% of missing values were discarded. Missing genotypes were imputed based on random sampling from marginal allele distributions using the R package synbreed (Wimmer et al. 2012). After quality filtering, 4781 evenly spaced SNPs were retained to compute the genomic relationship matrix **G**.

### Phenotypic analysis

A weighted two-stage approach was used for the analysis of phenotype data. In the first stage, each trial was individually analyzed to account for design factors and spatial field variation. In the second stage, spatially adjusted testcross means from the first stage were used to compute adjusted line means across testers and environments.

For the analysis of each trial, we applied a novel spatial method that adjusts for all types of field trends in a single modeling step by fitting a smoothed surface (Rodríguez-Álvarez et al. 2018a). We used the same flexible spatial model to analyze the whole series of trials and all traits. Velazco et al. (2017) showed that this approach performs as well as the more elaborate spatial methods, which are typically based on a specific multi-step modeling for each trial and trait. The general spatial model used across trial–trait combinations is defined as

1 $$y_{ijkl} = \mu + \, \left( {L \times T} \right)_{i} + B_{j} + R_{k} + C_{l} + f\left( {r,c} \right)_{kl} + e_{ijkl}$$

where the plot observation *y*_*ijkl*_ was modeled by fitting fixed effects for: the overall mean (*μ*), the *i*th line × tester hybrid (*L *× *T*) and the *j*th block (*B*); and random effects for: the *k*th row (*R*) and *l*th column (*C*). The term *f*(*r*, *c*) is a smooth function of row (*r*) and column (*c*) plot coordinates representing the fitted spatial surface, which simultaneously accounts for global and local trends (see Rodríguez-Álvarez et al. 2018a; Velazco et al. 2017 for details). Finally, *e* is the random spatially independent residual representing measurement error in each plot. All random effects were assumed independent homoscedastic and normally distributed with zero mean.

Spatial analyses were implemented within the REML-based mixed model framework using the R package SpATS (Rodríguez-Álvarez et al. 2018b).

In the second stage, spatially adjusted testcross means from all trials were jointly modeled by

2 $$y_{ijk} = \mu + L_{i} + T_{j} + E_{k} + LT_{ij} + LE_{ik} + TE_{jk} + LTE_{ijk} + e_{ijk}$$

where in this case *y*_*ijk*_ represents the adjusted mean estimated by best linear unbiased estimation (BLUE) of the *i*th female line crossed with the *j*th tester in the *k*th environment, which was fitted by a main line genetic effect (*L*), a main tester effect (*T*), a main environmental effect (*E*) and all possible interactions between these effects. The residual followed *e*_*ijk*_ ~ *N* (0, **R**), where **R** is a diagonal matrix with elements equal to the squared standard errors of each mean *y*_*ijk*_ estimated by model (1) in the first stage (Frensham et al. 1997). The analysis using diagonal weights instead of the full genotypic covariance matrix from each trial is preferable in practice because of its computational efficiency and comparable results (Möhring and Piepho 2009; Welham et al. 2010). Since trials were considered random, all the interactions involving *E* were random. Heterogeneous variances for the latter effects were allowed to improve goodness of fit of the model in each trait, as evaluated by the Akaike information criterion. In this stage, the effects *L* and *T* were also taken as fixed. However, given that not all lines were testcrossed with all testers, the interaction effect *LT* was considered random to estimate line means across testers (Bernal-Vasquez et al. 2014).

### Prediction models

Different parental or GCA models based on BLUP were applied to predict breeding values of female lines from progeny performance. These models differed in the amount of pedigree and genomic information used for predictions.

The prediction models used in our study assume that available SNPs may not explain all additive genetic variances. The general model formulation can be defined as:

3 $${\mathbf{y}}_{{\mathbf{L}}} = {\mathbf{1}}\mu + {\mathbf{Zg}} + {\mathbf{e}}$$

where the vector **y**_**L**_ contains the BLUEs of line effects ($\hat{L}_i$) from model (2), **1** is a vector of ones with associated general mean *μ*, **Z** is a design matrix allocating line BLUEs to unknown genetic effects, **g** is the vector of total additive genetic effects and **e** is the vector of residuals. Random residuals were assumed **e** ~ *N* (0, **R**), where **R** is a diagonal matrix with elements equal to the squared standard errors of genotypic BLUEs from the second stage of phenotypic analysis. This matrix accounts for differences in precision of estimated line means.

Total additive genetic effects were assumed ${\mathbf{g}}\sim\;N(0,{\mathbf{K}}\sigma_{\text{g}}^{ 2} )$, where **K** is a combined kinship matrix exploiting pedigree and genomic information. This matrix is constructed as **K** = *w***A** + (1 − *w*)**G**_**s**_, where **A** is the numerator relationship matrix among lines computed from the full pedigree and **G**_**s**_ is a rescaled genomic relationship matrix based on the SNP data (see details below). The weighting factor *w* represents the fraction of total additive variance $(\sigma_{\text{g}}^{ 2} )$ that is not captured by markers, such that $w\sigma_{\text{g}}^{ 2}$ is the amount of residual polygenic variance explained by genealogical relationships. In this model, referred here as K-BLUP, the weight can take any value between 0 and 1. It should be noted that **G** and **A** are not orthogonal; therefore, the interpretation of *w* should be considered with caution.

For our study, a sequence of eight values of *w* from 0.1 to 0.8, with increments of 0.1, was explored to assess the impact on predictions. For the sake of comparison, the extreme cases *w* = 0 and *w* = 1 were also considered. Note that the latter case corresponds to the traditional pedigree-based model, A-BLUP, which relies only on familial information. Alternatively, assuming *w* = 0 results in the basic G-BLUP model (VanRaden 2008), where predictions are exclusively conditional on marker-based similarities.

An equivalent formulation of the K-BLUP model is as follows:

$${\mathbf{y}}_{{\mathbf{L}}} = {\mathbf{1}}\mu \, + {\mathbf{Zm}} + {\mathbf{Za}} + {\mathbf{e}}$$

where the total additive genetic effects in (3) are decomposed into a vector of genomic additive effects (**m**) and a vector of residual polygenic effects (**a**), such that **g** = **m** + **a**, with respective variances $\sigma_{\text{g}}^{ 2} =\sigma_{\text{m}}^{ 2} + \sigma_{\text{a}}^{ 2}$. In this model, both types of genetic effects are assumed mutually independent with distributions ${\mathbf{m}}\sim\;N(0,{\mathbf{G}}_{{\mathbf{s}}}\sigma_{\text{m}}^{ 2} )$ and ${\mathbf{a}}\sim\;N(0,{\mathbf{A}}\sigma_{\text{a}}^{ 2} )$, where $\sigma_{\text{m}}^{ 2} = \, \left( {1 - w} \right)\sigma_{\text{g}}^{ 2}$ and $\sigma_{\text{a}}^{ 2} = w\sigma_{\text{g}}^{ 2}$. This parametrization, referred in the rest of the article as AG-BLUP, presents two differences compared to the K-BLUP model: first, the AG-BLUP model requires the fitting of two relationship matrices (**A** and **G**) to combine pedigree and genomic information, while this information is condensed into a single matrix in K-BLUP; second, under AG-BLUP, the magnitude of *w* is driven by the data in order to maximize the (restricted) likelihood of the model, as opposed to pre-specified weights used within the K-BLUP method. The performance of AG-BLUP was also compared to that of the best predictive K-BLUP model.

The **G**_**s**_ matrix used in our models was rescaled to make it compatible with **A** in reference to the same base breeding population. The adjustment was based on fitting **G**_**s**_ to **A** by applying **G**_**s**_ = *a* + *b***G**, where **G** is the unscaled genomic matrix as computed with the first method of VanRaden (2008), and the parameters *a* and *b* are estimated by equating the average levels of inbreeding and the overall relationships in **A** and **G** (Vitezica et al. 2011; Christensen et al. 2012). The added constant *a* accounts for old relationships among non-genotyped ancestral lines in the pedigree, while *b* is a scaling term accounting for the reduction in the genetic variance of genotyped lines relative to the pedigreed base population (see Legarra et al. 2014 for details).

Given the variance components from prediction models, the narrow-sense heritability ($h^{ 2}$) of line means was obtained as: $h^{ 2}=\sigma_{\text{g}}^{ 2} /\left( {\sigma_{\text{g}}^{ 2}+\sigma_{\text{e}}^{ 2} } \right)$, where $\sigma_{\text{e}}^{ 2}$ is the residual variance comprising nonadditive genetic effects and true errors associated with mean line estimates.

Models in the second stage of phenotypic analysis as well as prediction models were fitted using the average information REML (AI-REML) algorithm as implemented in the mixed model package ASReml-R (Butler et al. 2017).

### Model validation

The quality of predictions from each model was evaluated using a fivefold cross-validation technique. We considered two different strategies for splitting the data into training set (TS) and validation set (VS). These strategies emulate different selection schemes: one based on within-family predictions (W-fam) and one based on among-family predictions (A-fam). In W-fam, 20% of lines from each full-sib family formed the VS, i.e., predicted lines belong to families that were (tested) in the TS. In A-fam, we sampled 20% of whole families to construct the VS, i.e., predicted lines belong to full-sib families that were not present in the TS. With this setting, we examined contrasting levels of genetic relatedness between TS and VS: high for W-fam and low for A-fam. Each splitting scenario was repeated 20 times using the same random seed throughout all models. The line BLUEs from phenotypic analysis (**y**_**L**_) were regarded as the realized genetic values of lines and used for validation of prediction models.

### Prediction quality evaluation

Model performances were assessed using multiple criteria. In this research, predictive ability was taken as the primary criterion for identifying the best prediction model. However, measures of empirical bias and accuracy were also considered, as they offer complementary information that should be taken into account when evaluating the predictive performance of models. Predictive ability was measured as the Pearson’s correlation (*r*_PA_) between predicted additive values ${\mathbf {\hat{g}}}$ and realized values **y**_**L**_ in the validation set. Bias of predictions was investigated by regressing **y**_**L**_ on ${\mathbf {\hat{g}}}$, where a coefficient of regression *b* = 1 designates an empirically unbiased predictor, while *b* < 1 indicates inflation of the variance of genotype predictions or overpredictions. The mean squared error of prediction (MSEP) from the linear regression was also measured as an indicator of prediction accuracy, which incorporates concepts of both bias and precision. The evaluation of models was based on average values over the 20 replicates when using within-family or across-family relatedness for prediction. Significance of pairwise differences in predictive ability among prediction models was assessed by the Hotelling–Williams *t* test (Steiger 1980), which is the appropriate test when comparing two correlations that are not independent as a result of sharing a common variable (**y**_**L**_ in our case).

## Results

### Heritability and effect of scaling genomic relationships

Table 1 presents the narrow-sense heritabilities (*h*^2^) for all traits estimated from BLUP models using only pedigree relationships, using only genomic relationships or combining both sources of information. We also show the influence of scaling the genomic matrix on the estimates of *h*^2^. In general, heritabilities varied from relatively low in grain yield to high in the case of plant height. Models using the combined matrix **K** increased *h*^2^ for most traits compared to A-BLUP and G-BLUP. These changes in heritability reflect the ability of the models to capture genetic variation in the breeding population. Note that the weight *w* used to construct **K** in each case was defined to maximize the likelihood, being the resulting K-BLUP model equivalent to fitting the model AG-BLUP. Finally, using the scaled genomic matrix **G**_**s**_ in G-BLUP and K-BLUP, increased heritability estimates for all traits relative to the heritabilities obtained using the conventional genomic matrix.

**Table 1:** Estimates of narrow-sense heritability for grain yield (GY), stay-green (SG), plant height (PH) and flowering time (FT) from prediction models using only the pedigree-based matrix (A-BLUP), using only the unscaled (**G**) or the scaled (**G**_**s**_) genomic matrix and using the combined **K** matrix (K-BLUP)

Model	GY		SG		PH		FT	
	G	G_s_	G	G_s_	G	G_S_	G	G_S_
A-BLUP (*w *= 1)	0.40	0.40	0.61	0.61	0.70	0.70	0.63	0.63
G-BLUP (*w* = 0)	0.28	0.34	0.41	0.46	0.72	0.77	0.62	0.67
K-BLUP (*w* = maxLL)^a^	0.43	0.46	0.56	0.59	0.78	0.82	0.72	0.76

^a^maxLL: weight that maximized the REML log-likelihood in each case; for GY: *w* = 0.54 with **G** and *w* = 0.48 with **G**_**s**_, for SG: *w* = 0.43 with **G** and *w* = 0.38 with **G**_**s**_, for PH: *w* = 0.19 with **G** and *w* = 0.16 with **G**_**s**_ and for FT: *w* = 0.34 with **G** and *w* = 0.29 with **G**_**s**_

### Impact of combining pedigree and genomic relationships on prediction quality

**Fig. 1:**
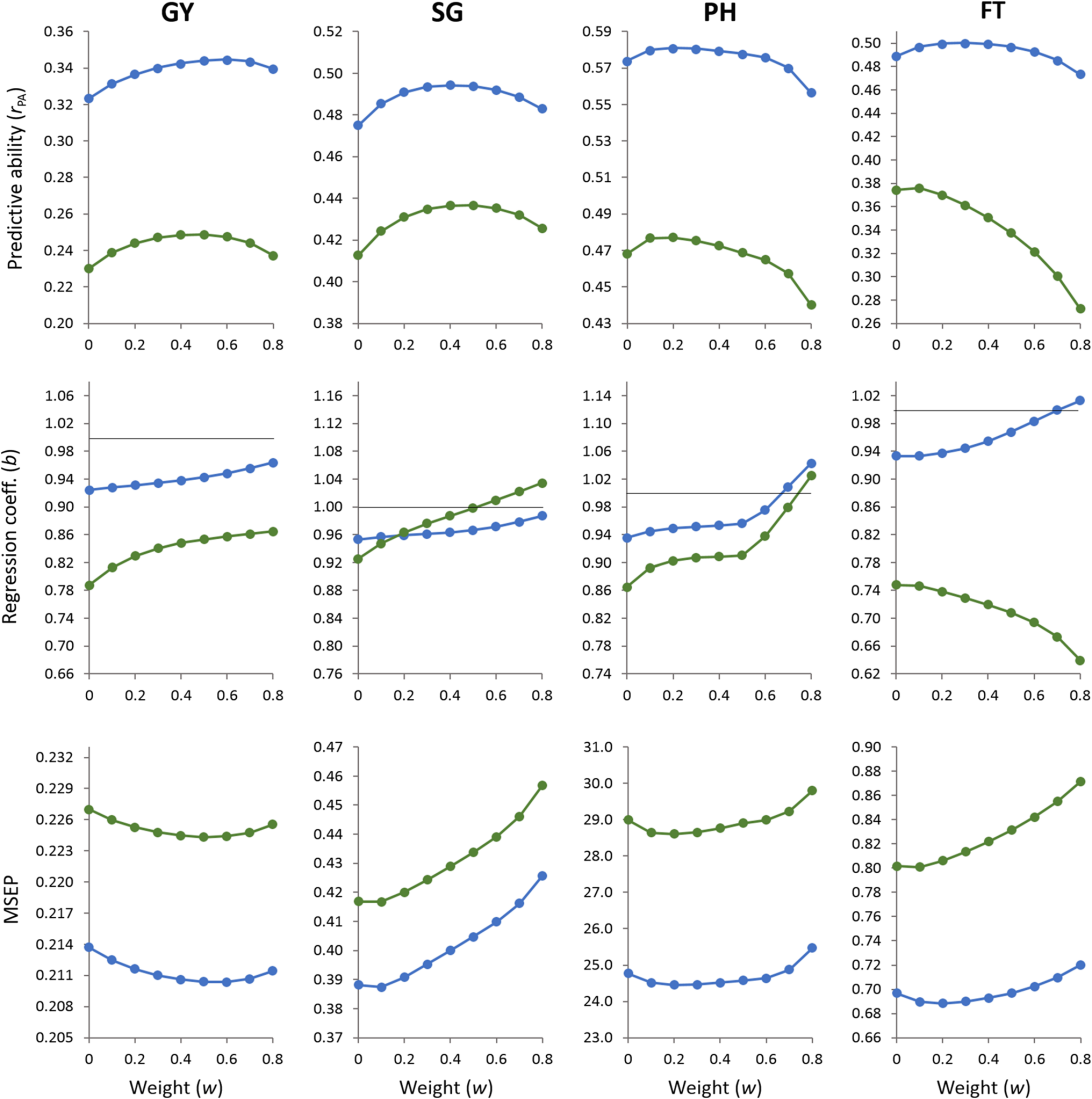
Predictive abilities, regression coefficients and MSEP from BLUP models using different weights (*w*) to construct the combined matrix **K** for grain yield (GY), stay-green (SG), plant height (PH) and flowering time (FT) predictions within (*blue*) and among (*green*) families. The weight *w* = 0 corresponds to the simple G-BLUP model. The *horizontal lines* indicate a regression coefficient *b* = 1 (color figure online)

Figure 1 shows the patterns of variation in prediction quality measures of BLUP models when using different weights (*w*) to construct the combined relationship matrix **K**.

In both prediction scenarios, predictive ability for GY and SG improved as the weight placed on pedigree information increased from *w* = 0 up to 0.4 ≤ *w* ≤ 0.6. The use of higher weights caused decreasing trends in *r*_PA_ for both traits. The largest relative increases in predictive abilities were observed for GY, with K-BLUP achieving 7% and 8% of improvement compared to G-BLUP (*w* = 0) for within- and among-family predictions, respectively. For PH and FT, *r*_PA_ was generally maximized by using lower weights (*w* ≤ 0.2) in both prediction scenarios. Any further increase in *w* slightly changed *r*_PA_ within families and was clearly detrimental for among-family predictions. Using a combined pedigree–genomic matrix provided more limited benefits for PH and FT, representing less than 2% of relative gains in *r*_PA_ over using only marker information.

In most cases, placing increasing weights on pedigree relationships reduced bias of predictions relative to predictions based on genomic relationships alone (*w* = 0). Moreover, the addition of genealogy information was more effective in reducing inflation of predictions when these were based on among-family relatedness. The only exception was observed for FT, where higher values of *w* gave more biased among-family predictions.

The use of increasing weights to form the **K** matrix reduced MSEP for GY, reaching the lowest values at *w* = 0.5 in both prediction scenarios. The opposite tendency was observed for the other traits, with higher accuracies obtained when more weight was assigned to genomic relationships and with accuracy of PH predictions being less sensitive to changes in *w*.

The optimal weighting factors for each trait and prediction scenario are given in Table 2. These weights were determined in order to maximize predictive ability of K-BLUP after examining the variation in *r*_PA_ over the set of candidate weights, as shown in Fig. 1. The best predictive weights based on cross-validation were generally similar to the weights that maximized the log-likelihood of the model for each trait in the entire breeding population (see footnotes to Table 1).

**Table 2:** Optimal weights (*w*) used to construct the combined matrix **K** for prediction of grain yield (GY), stay-green (SG), plant height (PH) and flowering time (FT) under within- and among-family prediction scenarios

Prediction scenario	GY	SG	PH	FT
Within families	0.6	0.4	0.2	0.2
Among families	0.5	0.5	0.2	0.1

### Performance of prediction models

Table 3 presents the measures of prediction quality from the BLUP models using pedigree and/or genomic information for all traits and both cross-validation scenarios. Independent of the prediction model, predictive abilities were significantly lower and predictions tended to be more biased when among-family information was used. The pedigree-based BLUP model, considered here as the benchmark, always gave the lowest *r*_PA_ and the highest MSEP. Statistically significant increases in *r*_PA_ were obtained by models exploiting genomic relationships alone or combined with pedigree in most cases. Exceptionally, the differences with A-BLUP became significant for GY predictions within families only when genealogy information was included in the genomic model. In general, the use of genome-wide information caused larger relative gains in *r*_PA_ for among-family predictions. The inclusion of marker-based relationships had a higher impact on improving *r*_PA_ for PH and FT, which were the traits with higher heritabilities. Models combining information from **A** and **G** consistently outperformed the basic G-BLUP in predictive ability. These improvements were statistically significant only for SG in both prediction scenarios. The highest predictive abilities were always achieved with K-BLUP, and this model exhibited the lowest MSEP in most situations. Moreover, including a residual polygenic component through the optimal **K** matrix produced the largest reductions in bias relative to G-BLUP in most cases. Although differences between AG-BLUP and K-BLUP were significant only for FT predictions among families, the latter model was slightly superior in predictive ability and prediction bias across all traits and both cross-validation schemes. The relative benefit of using the best predictive **K** matrix was more evident when predictions relied on among-family information.

**Table 3:** Mean values (and SD of 20 replicates) for predictive ability (*r*_PA_), relative increment of *r*_PA_ (∆ *r*_PA_), regression coefficient (Bias) and mean squared error of predictions (MSEPs) from BLUP models using different relationship matrices for grain yield (GY), stay-green (SG), plant height (PH) and flowering time (FT) prediction within and among families. The best values for each evaluation criterion are boldfaced

Trait	Quality criterion	A-BLUP	G-BLUP	AG-BLUP	K-BLUP^a^
*Within-family prediction*					
GY	*r*_PA_	0.299 (0.011)	0.323 (0.013)	0.339 (0.014)	**0.345** (0.014)
	∆ *r*_PA_(%)	0	8.1	13.4	**15.3**
	Bias (*b*)	**0.963** (0.040)	0.924 (0.045)	0.924 (0.043)	0.948 (0.045)
	MSEP	0.217 (0.002)	0.214 (0.002)	0.211 (0.002)	**0.210** (0.002)
SG	*r*_PA_	0.437 (0.010)	0.475 (0.007)	0.490 (0.009)	**0.494** (0.009)
	∆ *r*_PA_(%)	0	8.6	12.1	**13.0**
	Bias (*b*)	**0.988** (0.030)	0.953 (0.024)	0.952 (0.025)	0.963 (0.025)
	MSEP	0.514 (0.005)	**0.388** (0.004)	0.401 (0.005)	0.400 (0.005)
PH	*r*_PA_	0.420 (0.011)	0.574 (0.011)	0.579 (0.010)	**0.581** (0.009)
	∆ *r*_PA_(%)	0	36.6	37.8	**38.3**
	Bias (*b*)	**0.997** (0.032)	0.935 (0.024)	0.944 (0.023)	0.949 (0.021)
	MSEP	30.4 (0.4)	24.8 (0.5)	24.6 (0.5)	**24.4** (0.4)
FT	*r*_PA_	0.394 (0.015)	0.489 (0.011)	0.497 (0.014)	**0.500** (0.013)
	∆ *r*_PA_(%)	0	24.0	26.1	**26.8**
	Bias (*b*)	**0.964** (0.045)	0.933 (0.029)	0.937 (0.030)	0.944 (0.027)
	MSEP	0.774 (0.011)	0.697 (0.011)	0.693 (0.013)	**0.690** (0.013)
*Among-family prediction*					
GY	*r*_PA_	0.184 (0.037)	0.230 (0.027)	0.243 (0.028)	**0.249** (0.030)
	∆ *r*_PA_(%)	0	25.0	31.9	**35.1**
	Bias (*b*)	**0.858** (0.181)	0.788 (0.094)	0.828 (0.094)	0.853 (0.104)
	MSEP	0.231 (0.004)	0.227 (0.004)	0.225 (0.004)	**0.224** (0.004)
SG	*r*_PA_	0.365 (0.030)	0.413 (0.022)	0.426 (0.019)	**0.437** (0.016)
	∆ *r*_PA_(%)	0	12.9	16.7	**19.5**
	Bias (*b*)	1.007 (0.121)	0.925 (0.055)	0.958 (0.061)	**0.998** (0.050)
	MSEP	0.552 (0.014)	**0.417** (0.010)	0.432 (0.009)	0.434 (0.007)
PH	*r*_PA_	0.235 (0.044)	0.468 (0.022)	0.469 (0.021)	**0.477** (0.022)
	∆ *r*_PA_(%)	0	99.1	99.4	**102.9**
	Bias (*b*)	0.791 (0.155)	0.865 (0.051)	0.884 (0.056)	**0.902** (0.055)
	MSEP	35.0 (1.0)	29.0 (0.9)	28.9 (0.9)	**28.6** (0.9)
FT	*r*_PA_	0.156 (0.064)	0.374 (0.020)	0.352 (0.031)	**0.376** (0.023)
	∆ *r*_PA_(%)	0	139.6	125.2	**140.6**
	Bias (*b*)	0.448 (0.196)	**0.748** (0.047)	0.708 (0.066)	0.747 (0.051)
	MSEP	0.929 (0.042)	0.802 (0.019)	0.823 (0.030)	**0.801** (0.021)

^a^Using **K** matrices constructed with the specific optimal weights given in Table 2

## Discussion

Advances in genotyping technology have facilitated the implementation of genomic selection for several plant species. However, for some strategic cereal crops, especially sorghum, efforts are still needed to attain a full insight into the prospects of this genetic evaluation method. Here, we present a first comprehensive study addressing the potentialities of exploiting pedigree and genome-wide marker information to enhance prediction of parental BVs in sorghum. The idea of combining different kinship matrices for genomic prediction has also been introduced in the context of reproducing kernel Hilbert spaces regression using multiple kernels (de los Campos et al. 2010; Gianola and Schön 2016). This method provides a flexible framework, including the possibility of using nonlinear combinations of kinship matrices (Corrada Bravo et al. 2009; Gianola and de los Campos 2008). Our research is based on the BLUP method as it is easy to understand, compute and implement in available mixed model software. Moreover, several studies have shown that this method performs comparably to other models, such as the Bayesian alternatives, for prediction of complex quantitative traits in crops (e.g. Heslot et al. 2012; Wimmer et al. 2013).

Results showed that the use of genomic information consistently improved predictive ability and accuracy of prediction in sorghum relative to the classical pedigree-based method. This is expected because the **G** matrix accounts for Mendelian segregation of alleles, distinguishing between full-sib lines that are more or less related than expected due to random chance. Therefore, genomic estimated BVs of unphenotyped full sibs will reflect genetic differences caused by Mendelian sampling, while these sibs will have identical pedigree-based BVs reflecting only mid-parent genetic contributions. Similarly, specific pairs of lines from unrelated families may share more alleles than expected by chance and will have realized relationships different from zero. These features make the **G** matrix potentially more informative than **A** to better approximate genetic relationships between parental lines. The advantage of replacing pedigree by genomic-based similarities in prediction models has also been reported for other crops (e.g., Albrecht et al. 2011; Burgueño et al. 2012; Auinger et al. 2016). Less conclusive results regarding the relative value of **A** and **G** for prediction in sorghum were reported by Hunt et al. (2018). However, their study is not strictly comparable to the present research due to different predictive contexts. Hunt et al. (2018) used phenotypes of testcross progenies derived from crosses with a common tester. Consequently, the total genetic value of hybrids was predicted since general and specific combining ability effects could not be distinguished. In contrast, our study aimed to predict the additive genetic merit of parental lines given that the availability of testcross data using several testers allowed averaging out most dominance deviations. Moreover, predictions reported by Hunt et al. (2018) were based on separate analyses of individual trials, whereas we obtained across-environment predictions using multi-environment trial analysis.

Before combining pedigree and genomic information in our prediction models, marker-based relationships were adjusted to take into account the difference in scale between **A** and **G**. While relationships in **A** are defined in relation to the founder population of the pedigree, the reference population for relationships in **G** is automatically set to the genotyped individuals when current allele frequencies are used (VanRaden 2008; Hayes et al. 2009). Therefore, if the population genotyped has undergone drift or strong selection, which is usually the case in plant breeding programs, its average breeding value will be different and the genetic variance would be expected to be reduced relative to the founder breeding population (Legarra et al. 2014). Several studies using real and simulated data have shown that rescaling the **G** matrix improved predictions to a mild degree when not all individuals in the reference population were genotyped (Forni et al. 2011; Vitezica et al. 2011; Christensen et al. 2012). In contrast, the adjustment of **G** in our work, where genotypes were available for the entire training population, did not affect predictions (not shown). However, the rescaling did affect the scale of genomic variances, and thus changed heritability estimates (Table 1). These heritabilities derived from the adjusted **G** are thought to reflect a compatible scale for genomic- and pedigree-based estimates of genetic variability. Consequently, the correction of **G** provided a clearer theoretical framework for interpretation of parameter estimates when pedigree and SNP information are used simultaneously. Even though inconsistencies between the theory underlying classical polygenic models and the recent genomic approaches have received increasing attention in animal breeding, this topic has been largely overlooked in crop applications.

In order to investigate the benefits of adding pedigree information into G-BLUP, we used a kinship matrix based on a weighted linear combination of pedigree- and marker-based relationships, constructed as ${\mathbf{K}} = w{\mathbf{A}} + \, \left( {1 \, - w} \right){\mathbf{G}}.$VanRaden (2008) and Goddard et al. (2011) proposed deterministic methods to predict the appropriate *w* based on the error variance of the true genomic relationships or on the effective number of independent chromosomal segments, respectively. These methods and subsequent suggested alternatives provide variable theoretical estimates, and these can be substantially different from optimal data-dependent weights (Ilska et al. 2017). Here, we adopted an analytical approach where the best predictive *w* was empirically defined for each trait and cross-validation scenario after evaluating the changes in prediction quality over a sequence of candidate weights. The same approach was used applying a semi-parametric Bayesian method (Rodríguez-Ramilo et al. 2014) and in the context of multiple-trait prediction (Momen et al. 2017) in animal species. Our results in sorghum showed a general agreement between the best predictive weights based on cross-validation and the weights that maximized the fit of the model to the entire breeding population. This suggests that goodness of fit can be used as guiding tool to attain an optimal prediction model. Our study demonstrates, however, that the best fitting model may not produce the best predictive performance. This topic will be specifically addressed later in the present section when discussing the differences between K-BLUP and AG-BLUP.

In our research, the optimal weighting factor *w* varied across traits. This implies that the optimal similarity matrix, from a predictive perspective, is actually trait-specific. According to the optimal weights for PH and FT (Table 2), a large proportion of the total additive variation in these traits was captured by SNPs (between 80% and 90%). This is expected since our sorghum breeding population is highly structured, with strong family relationships and small effective population size. Consequently, the genetic variance explained by markers might not only be a result of SNPs located on causative genes or in LD at population level, but it might mainly depend on SNPs capturing familial relationships between lines (Habier et al. 2007). For GY and SG, lower amounts of additive genetic variance were explained by markers (between 40% and 60%) and higher weights on pedigree were generally required to optimize predictions. The lower levels of variance accounted for by markers may be due to the more complex genetic information driving these low-heritability traits, which was not totally captured by imperfect coverage of available SNPs. The higher importance of including pedigree information for GY and SG is reflected by the larger predictive improvements achieved from using K-BLUP instead of G-BLUP (Table 3). One reason may be that, when some markers are not in LD with QTLs, the addition of pedigree information contributes to capturing associations between causative alleles due to common ancestral identity, improving predictions. Furthermore, besides residual LD patterns explained by family structure, Jensen et al. (2012) pointed out that an additional polygenic component can also take into account potential LD across chromosomes. In agreement with our results, Liu et al. (2011) found that higher weights placed on pedigree information were required to optimize predictions of traits with lower heritabilities in dairy cattle. The use of trait-specific optimal weights has not only been recommended for genomic selection in animals (Liu et al. 2011; Gao et al. 2012), but also in wheat (Ashraf et al. 2016). In the latter study, identification of the optimal weighting terms was based on likelihood curves. The authors pointed out that differing weights had relatively small effect on improving the likelihood. We arrived at similar deductions when the weights were tuned to maximize predictive ability. Our results show that despite specific values of *w* were identified for each trait, a weighting factor between 0.4 and 0.5 would generally perform well across traits, with predictive performance being clearly sub-optimal only for FT predictions across families (Fig. 1). It should be considered, however, that these results are partly dependent on the marker density used.

In this paper, we compare a BLUP model blending pedigree and genomic information into a single matrix **K** with an equivalent model that fits **A** and **G** separately. While the latter approach has been previously used for genomic prediction in crops, the K-BLUP method had not been explored until now for plant breeding applications. Results show that the benefits of using K-BLUP instead of AG-BLUP were generally marginal but fairly consistent across traits and prediction scenarios. The predictive performance of AG-BLUP is expected to be sub-optimal compared to that of K-BLUP. The reason is that the weight used by AG-BLUP for prediction is the one that best fits the genotypes and phenotypes of the TS. However, this *w* is not necessarily an accurate estimator of the weight that optimizes prediction of phenotypes in the VS. Therefore, the likelihood-based *w* may contain some information that is only relevant for the reference lines, but with little predictive value for the validation lines. On the other hand, within the K-BLUP approach, *w* is empirically derived to optimize prediction of lines in the VS. Then, the resulting best predictive weight makes no direct reference to the TS data, but it seems to be closer to the *w* that best approximates the genetic variability among validation lines. This would explain why K-BLUP slightly increased predictive ability and reduced overprediction relative to AG-BLUP in all cases. Our results are consistent with those obtained by Ilska et al. (2017) in chicken, who found that the increased goodness of fit in the training set was accompanied by decreased accuracy and higher bias of predictions in the validation set.

Besides the difference in predictive performance, the fact that information from the two sources is conveyed by fitting a single relationship matrix provides additional benefits regarding model applicability. In our study, computational time was reduced by more than 35% when fitting **K** instead of **A** plus **G** (not shown). In addition, K-BLUP is likely to produce more stable results when using small training sets since fewer variance components have to be estimated. We applied relatively simple prediction models, but differences in computational burden and stability between AG-BLUP and K-BLUP are prone to increase when more elaborate models are used. For instance, when prediction models include interactions of genotypes with environmental factors or when multi-trait prediction is aimed. Finally, the use of a blended matrix prevents from potential convergence problems resulting from collinearity between pedigree-estimated genetic effects and genomic effects when **A** and **G** are fitted separately, which may also deteriorate the quality of predictions (Legarra et al. 2008).

In our study, the main criterion used to define the optimal weighting factor for K-BLUP was the predictive ability. Given that all selection candidates belong to the same generation, we considered that this was the criterion to maximize, since prediction bias should not be too strong. It is noteworthy that, in this research, the highest predictive ability and the smallest empirical bias were rarely achieved by the same model (Table 3). However, inflation or overprediction (*b* < 1) should also be considered when searching for the best predictor. For instance, inflation of genomic predictions can be detrimental for genetic gain since the genetic merit of new genotyped lines is overestimated when compared to older lines that have undergone testcross field evaluations. Our results revealed that including pedigree information through the blended matrix **K** always caused the largest reductions in the inflation produced by G-BLUP. Reduced bias of predictions obtained by increasing the weight on familial relationships has also been reported in animal genomic evaluations (Liu et al. 2011; Gao et al. 2012). In addition, the lowest MSEP was mostly achieved by K-BLUP in our research. The minimization of MSEP has been recommended as an appropriate evaluation criterion for the comparison of prediction models since it considers both bias and precision (Vitezica et al. 2011; González-Recio et al. 2014).

We evaluated model performance when within-family or across-family information was used for prediction. For all models, predictions were clearly better when full-sib relationships were exploited. The value of using information from close relatives for prediction in structured populations is consistent with the previous studies in animal and plant breeding (e.g., Wientjes et al. 2013; Schopp et al. 2017). Across traits, the decline in predictive ability was less marked for models including **G** when predictions were based on relationships among families (a mean decrease of 21% vs 40% for A-BLUP). This reflects the capacity of marker information to capture populational LD, which becomes particularly relevant when predictions rely on more distant genetic relatedness (Habier et al. 2007). As shown in Table 3, however, the relative benefit of using genomic information for among-family prediction was less evident for GY and SG. The latter finding emphasizes the contribution of additive genetic relationships and tempers that of LD for predicting traits with lower heritability.

## Conclusion

This paper provides a first empirical evidence based on sorghum breeding data that the use of genomic relationships alone, even with relatively low marker density, can give better predictions of parental BVs than the pedigree-based model. We also investigated how the use of different combinations of pedigree and genomic information affected prediction quality. Our results showed that using a kinship matrix integrating both sources of information yielded better predictive performance than G-BLUP for different traits and prediction scenarios. Identification of the optimal weighting factor used to combine familial- and marker-based relationships was driven by the search for maximizing predictive ability. Under this approach, the weight that optimizes predictions differed between traits. These weights were generally consistent with the weights that optimized model fitting to the entire dataset. The impact of including genealogy information to improve genomic predictions was stronger for traits with lower heritability, such as grain yield and stay-green. Findings of this paper might be relevant for other breeding programs with limited genotyping resources and when lowly heritable traits are the main targets of selection.
